# Divergent Effects of Laser Irradiation on Ensembles of Nitrogen-Vacancy Centers in Bulk and Nanodiamonds: Implications for Biosensing

**DOI:** 10.1186/s11671-022-03723-2

**Published:** 2022-09-26

**Authors:** Domingo Olivares-Postigo, Federico Gorrini, Valeria Bitonto, Johannes Ackermann, Rakshyakar Giri, Anke Krueger, Angelo Bifone

**Affiliations:** 1 Center for Neuroscience and Cognitive Systems, Istituto Italiano Di Tecnologia, Corso Bettini 31, 38068 Rovereto, Trento, Italy; 2 Molecular Biology Center, University of Torino, via Nizza 52, 10126 Turin, Italy; 3 Department of Molecular Biotechnology and Health Sciences, University of Torino, via Nizza 52, 10126 Turin, Italy; 4 Center for Sustainable Future Technologies, Istituto Italiano Di Tecnologia, via Livorno 60, 10144 Turin, Italy; 5 Institut Für Organische Chemie, Julius-Maximilians-Universität Würzburg, Am Hubland, 97074 Würzburg, Germany; 6 Wilhelm Conrad Röntgen Center for Complex Materials Research (RCCM), Julius-Maximilians University Würzburg, 97074 Würzburg, Germany

**Keywords:** Nanodiamonds, Polarization, Nitrogen-vacancy centers, Bulk diamond, Spin dynamics, Charge dynamics, Charge stability, Photoconversion, Nanomilling, ^13^C, Cells

## Abstract

Ensembles of negatively charged nitrogen-vacancy centers (NV^−^) in diamond have been proposed for sensing of magnetic fields and paramagnetic agents, and as a source of spin-order for the hyperpolarization of nuclei in magnetic resonance applications. To this end, strongly fluorescent nanodiamonds (NDs) represent promising materials, with large surface areas and dense ensembles of NV^−^. However, surface effects tend to favor the less useful neutral form, the NV^0^ centers, and strategies to increase the density of shallow NV^−^ centers have been proposed, including irradiation with strong laser power (Gorrini in ACS Appl Mater Interfaces. 13:43221–43232, 2021). Here, we study the fluorescence properties and optically detected magnetic resonance (ODMR) of NV^−^ centers as a function of laser power in strongly fluorescent bulk diamond and in nanodiamonds obtained by nanomilling of the native material. In bulk diamond, we find that increasing laser power increases ODMR contrast, consistent with a power-dependent increase in spin-polarization. Conversely, in nanodiamonds we observe a non-monotonic behavior, with a decrease in ODMR contrast at higher laser power. We hypothesize that this phenomenon may be ascribed to more efficient NV^−^→NV^0^ photoconversion in nanodiamonds compared to bulk diamond, resulting in depletion of the NV^−^ pool. A similar behavior is shown for NDs internalized in macrophage cells under the typical experimental conditions of imaging bioassays. Our results suggest strong laser irradiation is not an effective strategy in NDs, where the interplay between surface effects and local microenvironment determine the optimal experimental conditions.

**Supplementary Information:**

The online version contains supplementary material available at 10.1186/s11671-022-03723-2.

## Introduction

Negatively charged Nitrogen-Vacancy centers (NV^−^) can be used as probes for ultrasensitive detection of magnetic [1–3] and electric fields [4–6], temperature [7, 8], as well as electron and nuclear spins at the nanoscale [9, 10]. NV^−^ centers can be optically polarized and readout with irradiation of laser light at room temperature and at the Earth’s magnetic field [11–13], thus enabling applications in biomedical assays [14–17], including intracellular thermometry [7, 18, 19], optical magnetic imaging in living cells [2, 20, 21] or optical magnetic detection of single-neuron action potentials [22, 23]. Moreover, optical pumping of NV^−^ centers has been proposed as an alternative to dynamic nuclear polarization (DNP) for the hyperpolarization of nuclear spins inside or outside the diamond lattice [24–26].

These applications rely on ensembles of NV^−^ centers in the vicinity of the diamond surface [27, 28]. Due to their large surface area, NV-rich fluorescent nanodiamonds (NDs) are promising candidates for these applications [7, 15, 21]. Moreover, NDs are biocompatible, and [29–31] can be internalized in living cells, thus probing the microenvironment in subcellular compartments [32, 33]. Importantly, their surface can be functionalized to target specific cells or proteins [29–31]. Furthermore, ^13^C-enrichment of NDs can improve hyperpolarization efficiency, quantum sensing and magnetic resonance signal enhancement [34].

Several techniques have been proposed for the production of NDs, including nanomilling [35] of bulk diamond, detonation [36, 37] and high-power laser ablation [38, 39]. Detonation NDs tend to be small (5–10 nm) and rich in impurities [36]. High-power laser ablation can produce fluorescent NDs in a single-step process [38, 39], but the yield is still insufficient for practical applications. Conversely, nanomilling of bulk diamond makes it possible to control concentration of defects and NV centers, as well as particle size, with good production yield [40].

A larger surface area increases exposure of shallow NV centers to the external environment, thus improving sensitivity and potentially promoting polarization transfer to molecules outside the diamond surface. However, surface effects tend to favor the neutral charge state NV^0^ [41], which does not present useful spin properties [11–13]. As a result, a larger concentration of NV^0^ in NDs compared to the starting material is often observed [41].

Laser light used to polarize and interrogate NV^−^ centers can also induce charge conversion between NV^−^ and its neutral form NV^0^ [27, 42–47]. Photoconversion depends on the presence of nitrogen defects and surface acceptor states, and thus both NV^−^ → NV^0^ and NV^0^ → NV^−^ photoconversion routes have been observed in different diamond samples [13, 43, 44]. Recently, we have shown that increasing laser power can substantially enhance the availability of shallow NV^−^ in nanostructured, ^15^ N-implanted ultrapure CVD diamond [48]. However, it is unclear whether a similar strategy may be advantageous in NDs, and here we test this hypothesis in NDs of various sizes obtained by nanomilling of highly fluorescent ^13^C-enriched diamond. Fluorescence spectra and optically detected magnetic resonance (ODMR) spectra were acquired at different laser powers for NDs of 156 nm and 48 nm, and for the native bulk diamond, as a direct comparison. Additionally, we internalized NDs in macrophage cells to study the effects of laser power under the typical conditions of a bioassay and in the cellular environment. Experiments were performed with a house-built wide-field microscope at sub-micrometric spatial resolution (1 pixel corresponding to 160 nm) to account for the intrinsic heterogeneity in NDs behavior and to study the microenvironment in different cellular compartments. Our findings highlight the importance of surface effects on photoconversion and provide useful information on the optimization of experimental conditions for biosensing or polarization transfer applications involving fluorescent NDs.

## Materials and Methods

### Samples

**Fig. 1:**
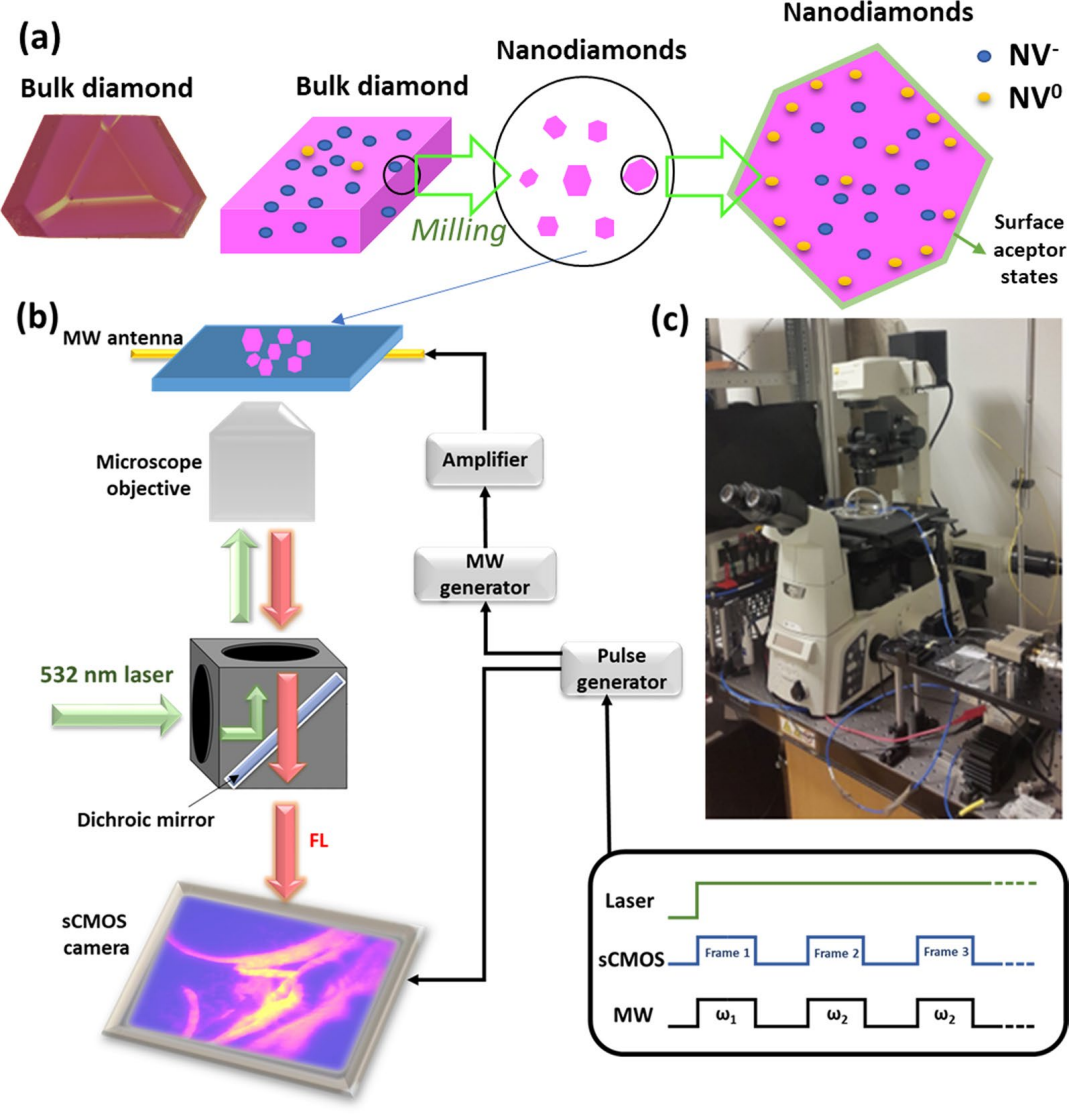
Preparation of NDs and schematic of the wide-field ODMR set-up. **a** NDs, 156 nm and 48 nm in size were obtained by milling bulk, ^13^C-enriched HPHT fluorescent diamond. **b** Schematics of the inverted wide-field ODMR microscope. Green laser light is focused onto the sample. The fluorescence is collected by the objective through a dichroic mirror to the high-sensitivity CMOS camera. The image shows an actual fluorescence image of NDs deposited on a glass slide. MW delivery is obtained with a loop adjacent to the sample. The panel shows the temporal diagram of the CW-ODMR experiment. The laser is kept ON during the entire measurement, while the camera detects the fluorescence image synchronously with the MW irradiation. **c** Photo of the wide-field ODMR microscope set-up

Bulk ^13^C-enriched diamond grown by the high-pressure high-temperature (HPHT) technique was acquired from ElementSix. These samples have a concentration of NVs of ≈10 ppm, and ^13^C enrichment ranged from 5 to 10% (≈$5*{10}^{4}$—${10}^{5}$ ppm), depending on the position within the diamond stone; concentration of substitutional nitrogen (P1 centers) was approximately 200 ppm. The fraction of ^13^C was provided by the vendor, while the concentrations of nitrogen and NV centers were estimated through spectroscopic measurements on the bulk diamond before milling [27]. Attrition milling was used to prepare samples with varying size distribution with a procedure adapted from [49]. The whole process is exemplified in Fig. 1a. The sample material (62 mg) was added to a stainless-steel milling cup, which was filled up to one third of its height with 5 mm-diameter stainless-steel milling balls. The bottom third of the milling cup was then filled with isopropanol that had been dried using molecular sieve. The sample was milled for six hours at 50 swings per second. At the end of the process, the sample contained a significant amount of metallic debris caused by the milling. To clean the diamond material, the sample was flushed out of the milling cup with distilled water into a 250 ml round bottom flask. The remaining steel balls were removed using a magnet. To dissolve metallic impurities, 50 ml of concentrated hydrochloric acid was added. The mixture was stirred overnight at room temperature. After settling, the supernatant was decanted and 50 ml 96% of sulfuric acid was added. The resulting mixture was heated to 120 °C bath temperature without a reflux condenser to remove any remaining isopropanol. After one hour, a reflux condenser was attached to the flask and 20 ml of nitric acid (65%) slowly added while monitoring the reaction mixture carefully to prevent a violent reaction. It is important to note that any remaining isopropanol might violently react with concentrated nitric acid, thus it is important to remove it carefully before adding HNO_3_! The solution was stirred overnight at 120 °C. After the solution had cooled down to room temperature, the supernatant acid mixture was removed using a pipette and the solution was transferred to centrifugation tubes. To wash the diamond material, centrifugation at 15,000 rpm was used over one hour to settle the diamond material at the bottom. The supernatant was removed and replaced with distilled water. The diamond material was then dispersed using sonication. This process was repeated until the supernatant showed a neutral pH. SEM images at different magnifications of milled fluorescent nanodiamonds before size separation by centrifugation can be found in Additional file 1: Fig. S1, together with the corresponding Raman Spectra (Additional file 1: Fig. S2). After reaching neutral pH, centrifugation was used to separate the particles by size. Two fractions were selected, corresponding to the biggest and the smallest particles in the suspension, and their size distribution measured using dynamic light scattering (DLS), giving a median value of the volume distribution (D50 value) of 156 nm and 48 nm, respectively (Additional file 1: Fig. S3 and S4). Finally, NDs were suspended in deionized water and stored in glass vials.

Prior to the FL and ODMR experiments, suspensions of NDs were sonicated, and 10 µL were deposited either on a circular glass slide or in an Ibides (Ibidi GmbH, Planegg/Martinsried, Germany) and allowed to dry. In a separate set of experiments, NDs were internalized into cells following the procedure described below.

### Cell Line

Murine (RAW 264.7) cell line was purchased from American Type Culture Collection (ATCC LGC Standards, Sesto San Giovanni, Italy) and cultured in DMEM supplemented with 10% (v/v) of fetal bovine serum (FBS), 2 mM L-glutamine, 100 U/mL penicillin and 100 μg/mL streptomycin at 37 °C in a humidified atmosphere with 5% CO_2_.

### In Vitro NDs Uptake Experiments

For uptake experiments, RAW 264.7 cells were seeded in an Ibidi at a density of 3 × 10^4^ cells/ well and incubated at 37 °C for 24 h, to allow them to adhere to the slide surface. Incubation of cells with NDs (size 156 nm and 48 nm) was performed for 24 h at 37 °C in a humidified atmosphere with 5% CO_2_. At the end of the incubation, cells were washed three times with phosphate buffered saline (PBS) and fixed in 4% paraformaldehyde (PAF) at room temperature for fifteen minutes.

### Confocal Microscopy

Cells were rinsed twice with PBS and permeabilized with 0.1% Triton in PBS for ten minutes. Actin filaments were stained with phalloidin fluorescein isothiocyanate (FITC) (Sigma) for thirty minutes at room temperature. After washing twice with PBS, nuclei were counterstained with 4′,6-diamidino-2-phenylindole (DAPI). Coverslips were mounted with a glycerol/water solution (1/1, v/v). Observations were conducted under a confocal microscopy (Leica TCS SP5 imaging system) equipped with an argon ion and a 561 nm DPSS laser. Cells were imaged using a HCX PL APO 63 × /1.4 NA oil immersion objective. NDs were excited by 561 nm laser, while the emission was collected in the 570–760 nm spectral range. Phalloidin was imaged using 458 nm laser and the emission collected in the 498–560 nm range. DAPI was imaged using 405 nm laser and the emission was collected in the 415–498 nm range. Image analysis was performed using ImageJ software.

### FL and ODMR Experimental Setup

Full fluorescence spectra of the NVs were acquired with a confocal microRaman setup (LabRam Aramis, Jobin–Yvon Horiba), equipped with a DPSS laser (532 nm) and an air-cooled multichannel CCD detector in the window range between 535 and 935 nm.

Wide-field ODMR imaging was performed with a modified Nikon Ti-E inverted wide-field microscope (Fig. 1b,c) equipped with a microwave channel and a high-sensitivity CMOS camera (Hamamatsu ORCA-Flash4.0 V2) [39]. A 532 nm continuous-wave laser (CNI laser, MGL-III-532/50mW) was used as excitation source, delivering on the sample a power of ~ 30 mW through a 40X (NA = 0.75 and working distance of 0.66 mm) refractive objective. The laser power was modulated by inserting neutral density filters on the optical path. To avoid backscattering of laser light, we used a custom made dichroic beamsplitter. FL was collected in a spectral window ranging from 590 to 800 nm.

The microwave (MW) field source was a WindFreak MW generator (SynthHD v1.4 54 MHz-13.6 GHz); the signal was amplified by a Mini-Circuits ZVE-3 W − 83 + 2 W amplifier. The MW irradiation was delivered to the sample through a MW single loop terminated with a high-power MW damper.

The temporal sequence of the experiment (camera acquisition and MW delivery) was controlled by a SpinCore 100 MHz TTL generator (Model: TTL: PB12-100-4 K). Finally, the image acquisition was processed with the Nikon NIS-Elements Advanced Research software and analyzed with Fiji software.

The home-built instrument used for these experiments makes it possible to extract ODMR spectra pixelwise with sub-micrometric spatial resolution, thus enabling analysis of heterogeneously distributed samples. Indeed, the deposition procedure can result in a non-uniform distribution of NDs, with region-dependent concentration and size of aggregates. NDs uptake from cells is also inhomogeneous, and cells with varying amount and clustering of NDs inside were observed.

### ODMR Technique Description

This modified wide-field microscope was used to perform spatially resolved continuous-wave ODMR (CW-ODMR), a technique that probes the sublevel structure of the ground state. Hence, ODMR spectra are sensitive to changes in level structure induced by external magnetic or electric fields, interactions with paramagnetic substances or temperature. ODMR is therefore useful to exploit the sensing properties on NV^−^ centers in diamond.

The ground state of the NV center consists of a spin triplet, with $\left|{g,m}_{s}=\pm 1\right.\rangle$ states upshifted by $2.87$ GHz with respect to $\left|{g,m}_{s}=0\right.\rangle$ state. Continuous irradiation with a 532 nm laser polarizes $\left|{g,m}_{s}=0\right.\rangle$ state thanks to non-radiative spin dependent transitions from the excited state through the metastable singlet states. Different laser power levels in the range 1–30 mW are used for this experiment. MW frequency is swept in the 2.75–3.00 GHz region at a fixed power output of 15 dBm to detect both the NV^−^ central resonances (centered at 2.87 GHz) and the ^13^C sidebands, generated by hyperfine interaction between the NV^−^ and a ^13^C nuclear spin in first neighbor position. When the MWs are resonant with $\left|{g,m}_{s}=0\right.\rangle \leftrightarrow \left|g,{m}_{s}=\pm 1\right.\rangle$ energy separation, the darker states $\left|g,{m}_{s}=\pm 1\right.\rangle$ become populated, and a drop in the fluorescence (a “dip” in the ODMR spectra) is recorded. Thus, the ODMR contrast provides a measure of spin polarization of the $\left|{g,m}_{s}=0\right.\rangle$ and of spin population transferred to $\left|{g,m}_{s}=\pm 1\right.\rangle$. However, the ODMR spectrum is also affected by the relative contribution from NV^0^ fluorescence, which is not modulated by MW and offsets background fluorescence. An increase in ODMR contrast may thus indicate increased polarization of NV^−^, or decreased concentration of NV^0^s. The sequence of laser and MW irradiation to perform the ODMR experiments is described in the box of Fig. 1b.

## Results and Discussion

**Fig. 2:**
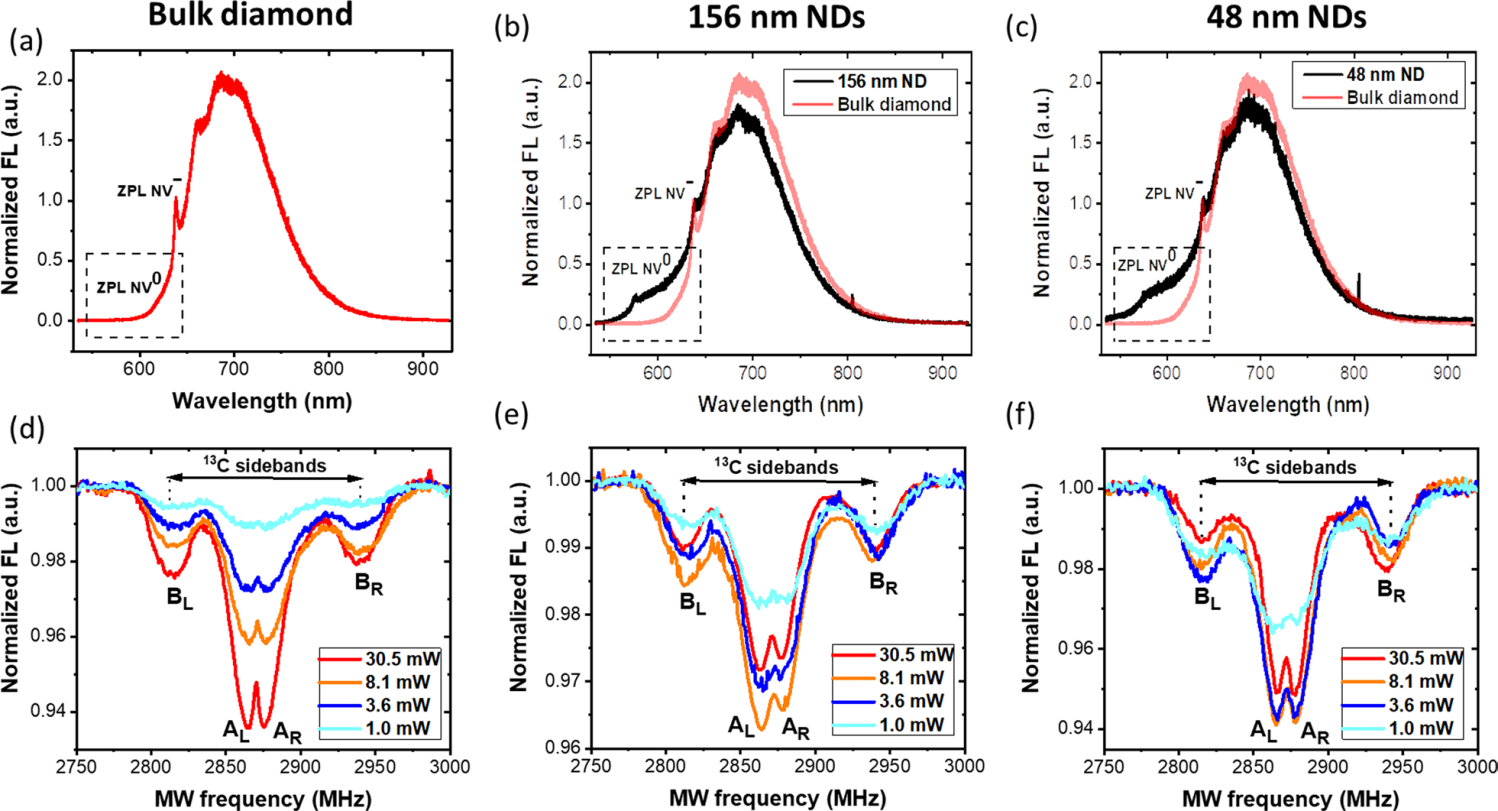
Fluorescence and ODMR spectra. Fluorescence and ODMR spectra for the **a**,**d** bulk diamond, **b**,**e** 156 nm and **c**,**f** 59 nm NDs, respectively. **a**,**b**,**c** NV^0^ and NV^−^ fluorescence spectra are characterized by ZPL lines at 575 nm (NV^0^) and 638 nm (NV^−^). Due to surface effects, NDs have a larger NV^0^ component than bulk diamond, where the NV^0^ band is practically undetectable. Panels **d**,**e**,**f** show ODMR spectra at different laser power with fixed MW power. In the ODMR spectra, the NV^−^ central lines (*A*_L_ and *A*_R_) provide a measure of the spin polarization of $\left|\mathrm{g},{\mathrm{m}}_{\mathrm{s}}=0\right.\rangle$ ground state. The MW range was set to 2.75–3 GHz to show the NV^−^ central lines and the ^13^C-coupled sidebands (*B*_L_ and *B*_R_). In bulk diamond, ODMR contrast increases with increasing laser power. Conversely, in the NDs, the ODMR contrast increases up to 8.1 mW and then, decreases at higher laser powers. Both FL spectra and ODMR spectra were acquired under continuous laser irradiation. The small peak at ~ 800 nm in the FL spectra is an artifact of the Raman spectrometer

Figure 2a–c shows the fluorescence (FL) spectra from the bulk diamond, the 156 nm and the 48 nm NDs. Each NV charge state is characterized by its fluorescence spectrum, with zero-phonon lines (ZPL) at 575 nm and 638 nm for the NV^0^ and NV^−^, respectively. In addition, a phonon sideband, peaked around 620 nm for the NV^0^ and 700 nm for the NV^−^, is observed, extending up to ≈ 850 nm in both cases. The FL spectra are normalized to the NV^−^ ZPL for comparison. Moreover, NDs showed a larger component from the NV^0^s with respect to the bulk diamond (represented as black and light red curves, respectively). This is consistent with the idea that surface effects favor the NV^0^ centers [44, 50], thus affecting the relative concentration of the different charge states.

Figure 2d–f shows the ODMR spectra of the bulk diamond and NDs at different laser powers (from 1 to 30.5 mW) at constant MW power of 15 dBm. The ODMR spectra show the NV^−^ central lines (*A*_L_ and *A*_R_) and two side bands (*B*_L_ and *B*_R_), the latter related to the hyperfine interaction between the NV^−^ spins and the ^13^C nuclear spins [51]. The ^13^C sidebands are separated from each other by ~ 130 MHz, consistently with previously reported values [34, 51–54]. For all samples, the linewidth of the resonance bands decreases with laser power, in agreement with the line-narrowing effect described by Jensen et al. [55]. Alongside with the increase in signal-to-noise (SNR) ratio, this phenomenon improves resolution of the NV^−^ strain-split doublet (central dips A_L_ and A_R_). We did not observe any detectable temperature effect on the position of the ODMR resonance that may be caused by absorption of MW or laser power [4].

**Fig. 3:**
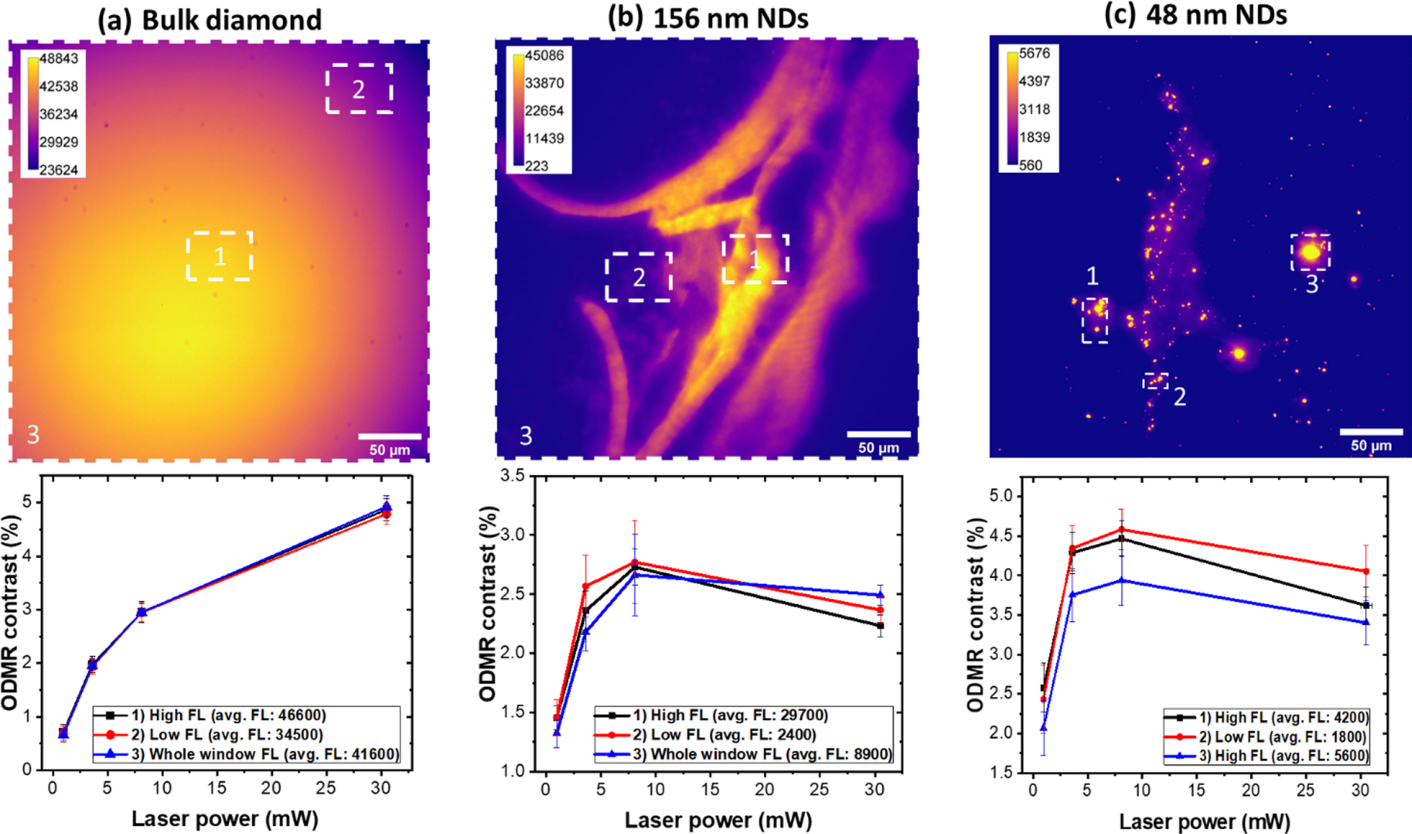
Comparison of ODMR contrast at different laser powers for bulk diamond and NDs. ODMR curves are extracted from three different regions of interest (ROIs) containing the bright spots that indicate presence of NV centers (see wide-field fluorescence images). As laser power increases, in bulk diamond the ODMR contrast increases, consistently with larger NV^−^ polarization levels (**a**). Conversely, in NDs of both sizes, the ODMR contrast increases up to 8.1 mW and decreases at higher laser powers, resulting in a non-monotonic behavior (**b**,**c**). The homogeneity of bulk diamond is reflected in the uniform values of ODMR contrast observed in the various ROIs with different levels of FL (values in the caption). On the contrary, NDs show non-uniform deposition and aggregation, resulting in a region-dependent ODMR contrast. 156 nm NDs were less inhomogeneous than the 48 nm NDs. The laser power dependence of the ODMR signal was similar in all the ROIs extracted, for both NDs sizes

In bulk diamond, a monotonic increase in ODMR contrast with laser power was observed, consistent with increasing NV^−^ polarization levels (Figs. 2d and 3a). Contrast was calculated as the mean value from the central resonances A_L_ and A_R_. Interestingly, in NDs, a non-monotonic behavior was observed, as shown in Fig. 3b, c. Up to 8.1 mW of laser power the ODMR contrast increases. At the highest power levels, the trend is the opposite, with a decrease in contrast systematically observed in different sample regions and for both ND sizes (Fig. 3b, c).

In principle, a reduction of the ODMR contrast in NDs observed at the highest laser power might result from the competition between optical and MW irradiation. We note that all experiments were performed at constant MW power, while laser power was varied. As a result, at the highest laser power, the spin system may be optically repolarized, with a net decrease in the observed ODMR contrast. However, this does not appear to be the case in this set of experiments. Indeed, the opposite effect is observed in the native bulk material, which has the same composition and concentration of nitrogen as the NDs, and was studied under identical experimental conditions. Moreover, the laser power density in our experiments is very low (up to 3 μΩ/μm^2^), and far from saturation at the MW power used. This is orders of magnitude smaller than laser power saturation levels previously reported [56, 57].

More likely, our observation can be explained in terms of different contributions of NV^0^ centers in the bulk and ND samples under the various experimental conditions explored here. In fact, we notice that both NV^−^ and NV^0^ signals are collected by the broad bandpass filter with a spectral window of 590–800 nm (see Methods). The ODMR contrast is defined as ${C}_{s}=\left({I}_{off}-{I}_{on}\right)/{I}_{off}$, where ${I}_{off}$ and ${I}_{on}$ are the NV^−^ FL intensities with MWs off- and on- resonance, respectively. However, the FL contains a contribution $\left({I}_{0}\right)$ from the NV^0^ centers, which is not modulated by MWs and reduces the contrast by a factor ${I}_{off}/\left({I}_{off}+{I}_{0}\right)$. This reduction in contrast is very different for bulk and NDs. In the bulk, the vast majority of the NV centers are negative, as shown in Fig. 2a, and remain stable under laser irradiation. Therefore, a stronger laser irradiation results in a better spin initialization and improved ODMR contrast, without impacting on the NV charges (I_0_ negligible). On the contrary, NDs tend to have higher relative concentrations of NV^0^ centers as a result of surface effects (Fig. 2b, c). Moreover, under laser irradiation, NV^−^ → NV^0^ photoconversion might be more efficient in NDs due to presence of surface acceptor states that can take a photoexcited electron from NV^−^ [44, 50]. Higher laser powers can then result in increasing relative concentrations of NV^0^ in NDs, and in a reduction in ODMR contrast. This phenomenon competes with NV^−^ polarization, which increases with laser power, resulting in the non-monotonic behavior shown in Fig. 3b, c. Therefore, in NDs there is an optimal laser power that should be used to prepare the NV^−^ spin states. Finally, we point out that the inhomogeneity in the NDs distribution, apparent in the FL images, together with the reduced stability of the NV^−^ charge state (Fig. 2b, c) can explain the lower SNR and the larger error bars affecting the ODMR contrast. Stabilization of the NV^−^ might improve the SNR, as well as shift the maximum of the curve of Fig. 3b, c to higher laser power.

A practical difficulty in assessing the properties of ensembles of NDs is the large variability of the optical response in heterogeneous samples [41, 58–60]. This has been ascribed to inter-aggregate interactions, size distribution and different efficiency in NV center initialization in ND aggregates [60]. To circumvent this problem, we resorted to use a wide-field fluorescence microscope to spatially resolve ODMR spectra in different parts of the sample. The heterogeneity in ND deposition is apparent in Fig. 3b, c, where the wide-field FL images clearly show inhomogeneous aggregation and concentration of NDs. Aggregation is particularly evident for the smallest NDs of 48 nm. ODMR contrast in these samples depends on the selected ROIs, characterized by different levels of FL (see caption of Fig. 3), with lower variability observed in the 156 nm NDs compared to the 48 nm NDs. Conversely, the ODMR contrast from the bulk diamond is uniform throughout the image. Despite region and sample dependent contrast, our data show consistent trends for NDs in different samples and ROIs, thus suggesting that the effect here reported is robust and reproducible.

**Fig. 4:**
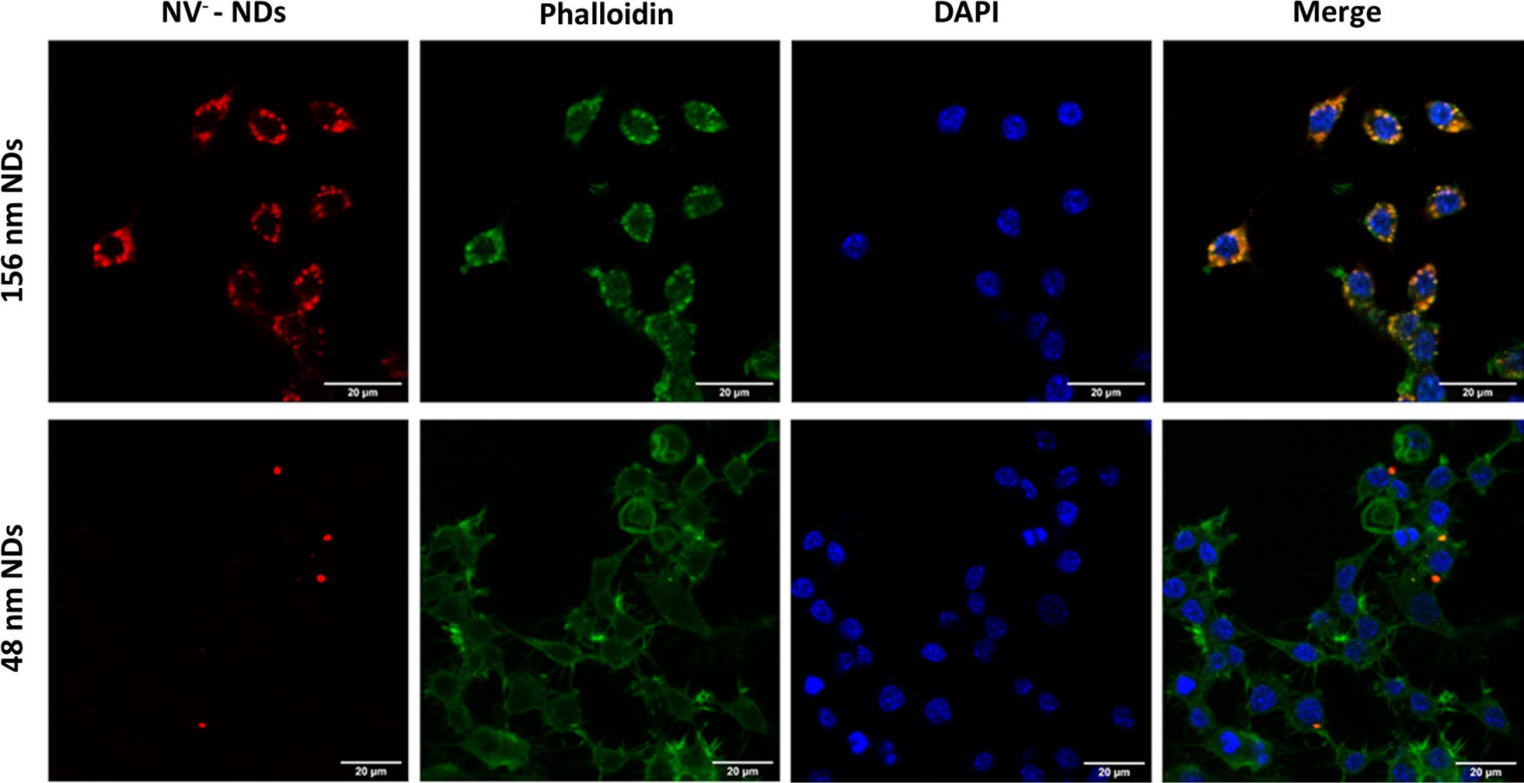
Confocal Microscopy images of RAW 264.7 cells incubated for 24 h with 48 nm and 156 nm NDs. After adding NDs (red) cells were stained with Phalloidin (green). Nuclei were counterstained with DAPI (blue). The “merge” figure demonstrates the internalization of the NDs (orange) within the cells. Cellular uptake of the 156 nm NDs is much higher than that of the 48 nm NDs. Scale bars = 20 µm. Representative images are shown

Charge dynamics and spin properties of NVs also depend on the ND microenvironment. To explore the effects described above in a typical bioassay, we incubated the NDs in cell cultures of macrophages (RAW 264.7). Internalization in macrophages is described in the methods and illustrated by the confocal images of Fig. 4. The composite panel on the right shows the NDs (orange) internalized in the cells, together with the actine filaments (green) and the nuclei (blue), for both 156 nm NDs (top row) and 48 nm NDs (bottom row). The images show that NDs accumulate in the cytoplasm without accessing the nuclei. The cells underwent a rinsing procedure to remove most of the non-internalized NDs that could otherwise contribute with a confounding background emission. Hence, the signals reported in these images correspond to a large extent to internalized NDs.

The concentration of the 48 nm NDs in the native suspension appears to be too low to provide sufficient fluorescence signal after incubation with cells to measure spatially resolved ODMR spectra. Hence, in the following, we focus only on the 156 nm NDs.

**Fig. 5:**
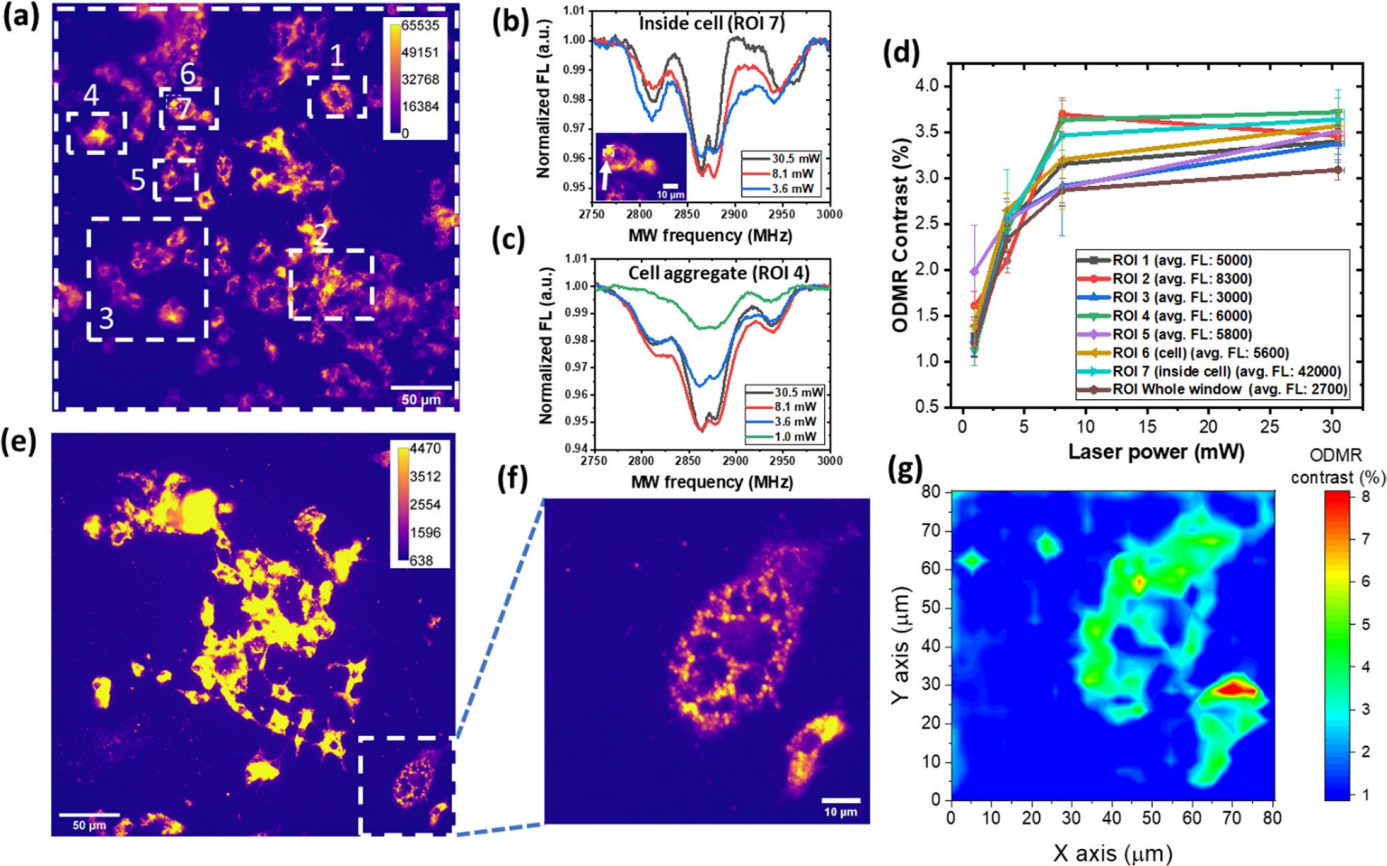
ODMR contrast of NDs internalized in cells. **a** Fluorescence image of 156 nm NDs internalized in macrophage cells; the white square delineates the ROIs from which signals were extracted. NDs internalized within cells show inhomogeneous aggregation and concentration, resulting in a region-dependent FL values. ODMR spectra presenting the NV^−^ central lines (*A*_L_ and *A*_R_) and the ^13^C sidebands (*B*_L_ and *B*_R_) at different laser power with constant MW power are shown in **b** for a single cell, and in **c** for a cellular aggregate. **d** ODMR curves corresponding to the ROIs of panel (**a**). The caption reports the average value of fluorescence intensity in each ROI. ODMR contrast steadily increases to 8.1 mW, while at higher laser powers it shows a plurality of behaviors. Small differences in the curves are uncorrelated with the average FL intensity and are supposedly related to local environment. **e** A color-saturated fluorescence image of 156 nm NDs internalized in cells. The selected ROI **f** shows a cell with its different compartments and its nucleus. **g** Map of the distribution of ODMR contrast within the cells of (**f**). Contrast varies regionally, with an average value of 4.5%

In Fig. 5a we show the fluorescence images using ROIs of different size and location in the cell culture incubated with the 156 nm NDs. Representative examples of ODMRs curves extracted from single-cell ROIs (~ 6 × 6 µm) or cell-aggregates ROI (from ~ 40 × 40 µm to ~ 100 × 100 µm) are shown in Fig. 5b and c, respectively. Despite some line broadening, compared to the bare NDs, the NV^−^ central bands and ^13^C sidebands can still be resolved. As in the case of bare NDs, the linewidth of the resonances decreases with increasing laser power, while the SNR increases, therefore improving the resolution of the NV^−^ doublet. Also in this case, we do not observe any variation depending on temperature (i.e., no shift of central resonances), indicating negligible heating by MW absorption by water in the cells or in the biological environment.

Figure 5d shows the evolution of ODMR contrast with laser power for different ROIs. Interestingly, the ODMR contrast behavior at high laser power is more variable than in the bare NDs of Fig. 3b, c, ranging from a small reduction, to a plateau-like behavior or even a slight increase. We speculate that this wider heterogeneity may be due to differences in the microenvironment, and particularly to the different pH of various cellular compartments (e.g., pH ≈ 5 in lysosomes compared to pH ≈ 7.2 in the cytoplasm).

Indeed, pH can affect the functional groups at the ND oxidized surface (carboxylic acids, ketones, alcohols, esters, etc.), thus changing the properties and charge stability of shallow NV centers [61, 62]. At low pH, e.g., carboxylates will be protonated to a much higher extent than under physiological conditions at pH ≈ 7.2, with potential effects on charge state of nearby NV centers. We stress that this hypothesis, while plausible, require further investigation.

As a faster method to assess the heterogeneity of the ODMR spectra in cells (Fig. 5e, f), we set up a simple procedure. Specifically, we acquired two fluorescence images, with MW on- and off- resonance. Taking their difference and then normalizing the fluorescence signals pixelwise, it is possible to reconstruct an ODMR contrast image (Fig. 5g). In these ODMR contrast maps, an average ≈ 4.5% contrast is observed. Moreover, parts with a high (red) and a low (blue) ODMR contrast can be resolved within the cell.

This technique, much faster that the acquisition of the full ODMR spectrum, could prove useful in fast mapping of ODMR, thus paving the way to real-time, spatially resolved imaging of temperature [7, 63], magnetic fields [21, 30, 64] or paramagnetic centers in live-cell bioimaging assays [45, 65]. We note that, being based on the ratio between FL levels and not on the absolute FL values, this technique is more affected by noise fluctuations. Here, we applied a 1% contrast threshold in the ODMR contrast mapping to minimize the contributions of undesired spurious signal.

## Conclusion

To summarize, we have studied fluorescence and optically detected magnetic resonance in fluorescent NDs obtained by nanomilling and in the native bulk material. A wide-field fluorescence microscope was used to address the problem of a potentially heterogeneous response of NDs within samples.

Our results show markedly different dependencies on laser power for the ODMR contrast in bulk diamond and NDs. While contrast and NV^−^ spin polarization steadily increase with laser power in the bulk diamond, with little to no variation across the sample, a more complex and heterogeneous behavior is observed in the NDs. In bare as-deposited NDs, we report a decrease in the ODMR contrast at the highest laser power explored here, possibly reflecting a more efficient NV^−^ → NV^0^ photoconversion compared to the bulk, and implying a laser-induced depletion of the pool of NV^−^ centers. The non-monotonic behavior in NDs is likely to be determined by the interplay between spin and charge dynamics under continuous laser illumination. For NDs internalized in cells we observed a qualitatively similar trend as in the bare NDs, with a different, more variable behavior at the highest laser power, suggesting that the cellular environment may play a role in the dynamics of NV charges, perhaps due to the different pH or to surface interactions with different proteins in the cytosol.

In conclusion, the large exposed surface area of NDs is greatly beneficial for sensing applications, *e.g.,* in bioimaging assays, but the effects of surface states and surface interactions on the NV charge stability and photoconversion dynamics should be taken into account. Increasing laser power in the native bulk diamond increases ODMR contrast, a measure of the spin polarization of the ensemble of NV^−^. Conversely, in NDs, surface effects limit the benefits of stronger laser power due to photoconversion between different charge states. The effects reported here highlight a trade off in the use of NDs for sensing and polarization transfer applications.

## Supplementary Information

**Additional file 1:** SEM, raman spectroscopy and dynamic light scattering of milled fluorescent nanodiamonds.

## Data Availability

The datasets used and/or analyzed during the current study are available from the corresponding author on reasonable request.

## Abbreviations

NDs: Nanodiamonds
NV: Nitrogen-vacancy center
NV^−^: Negatively charged nitrogen-vacancy center
NV^0^: Neutral charged nitrogen-vacancy center
P1: Substitutional nitrogen center
CW-ODMR: Continuous-wave-optically detected magnetic resonance
FL: Fluorescence
ROI: Region of interest
MW: Microwave
ZPL: Zero-phonon line
CVD: Chemical vapor deposition
HPHT: High-pressure high-temperature
FBS: Fetal bovine serum
PBS: Phosphate buffered saline
FITC: Fluorescein isothiocyanate
DAPI: 4′,6-Diamidino-2-phenylindole
WD: Working distance
NA: Numerical aperture
