# Supplementary material for: Divergent Effects of Laser Irradiation on Ensembles of Nitrogen-Vacancy Centers in Bulk and Nanodiamonds: Implications for Biosensing
Source: Nanoscale Res Lett. 2022 Sep 26;17:95. doi: 10.1186/s11671-022-03723-2 (PMC9512947; doi:10.1186/s11671-022-03723-2)
Supplement: Supplementary file 1 — Additional file 1: SEM, raman spectroscopy and dynamic light scattering of milled fluorescent nanodiamonds. [file 11671_2022_3723_MOESM1_ESM.docx]

**Supplementary Information**

Divergent effects of laser irradiation on ensembles of nitrogen-vacancy centers in bulk and nano-diamonds: implications for biosensing

Domingo Olivares-Postigo^1,2,3^*, Federico Gorrini^2,4^, Valeria Bitonto^3^, Johannes Ackermann^5^, Rakshyakar Giri^1^, Anke Krueger^5,6^ and Angelo Bifone^2,3,4^

^1^ Center for Neuroscience and Cognitive Systems, Istituto Italiano di Tecnologia, Corso Bettini 31, 38068 Rovereto, Trento, Italy

^2^ University of Torino, Molecular Biology Center, via Nizza 52, 10126, Torino, Italy

^3^ University of Torino, Department of Molecular Biotechnology and Health Sciences, via Nizza 52, 10126, Torino, Italy

^4^ Istituto Italiano di Tecnologia, Center for Sustainable Future Technologies, via Livorno 60, 10144, Torino, Italy

^5^ Institut für Organische Chemie, Julius-Maximilians-Universität Würzburg, Am Hubland, 97074 Würzburg, Germany

^6^ Wilhelm Conrad Röntgen Center for Complex Materials Research (RCCM), Julius-Maximilians University Würzburg

97074, Germany

* domingo.olivares@iit.it

**Table of contents**

1. SEM of milled fluorescent ND ………………………………………p. 2
2. Raman spectroscopy of milled fluorescent ND ………………………………………p. 3
3. Dynamic light scattering of milled fluorescent ND ………………………………………p. 3
4. **SEM of milled fluorescent ND**

|  |  |
| --- | --- |
|  |  |
|  | ***Figure S1 \|*** *SEM images of milled fluorescent diamond (fND) before size separation by centrifugation and at different magnifications*  *The samples were prepared by drop-coating on silicon substrate with an aqueous solution of the milled fND.*  *Scale bars: 100 nm (upper left), 2 µm (upper right), 200 nm (lower left)* |

1. **Raman spectrum of milled fluorescent ND**


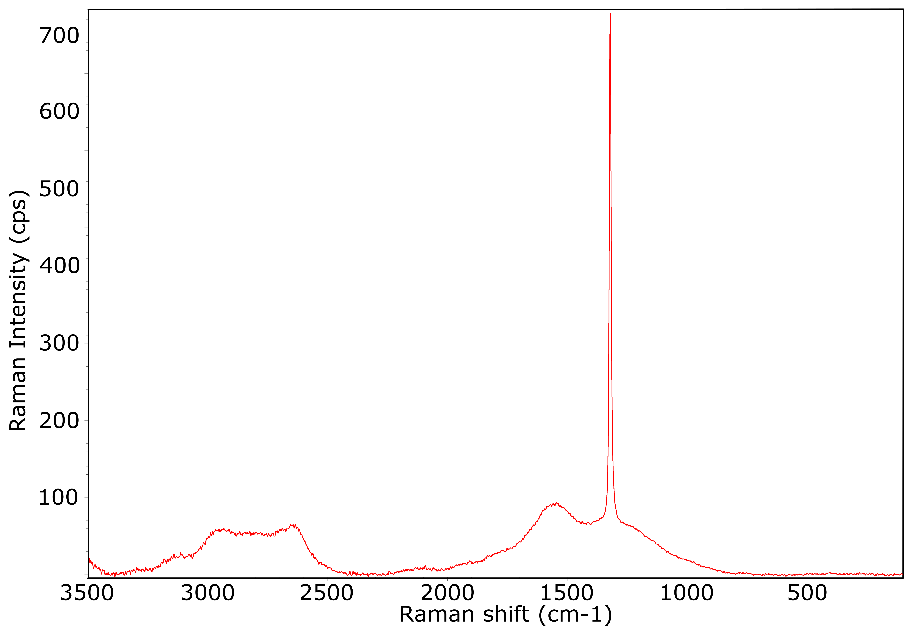


***Figure S2 |*** *Raman spectrum of milled fND using laser excitation at a wavelength of 445 nm.*

1. **Dynamic light scattering of aqueous colloids of milled fluorescent ND**


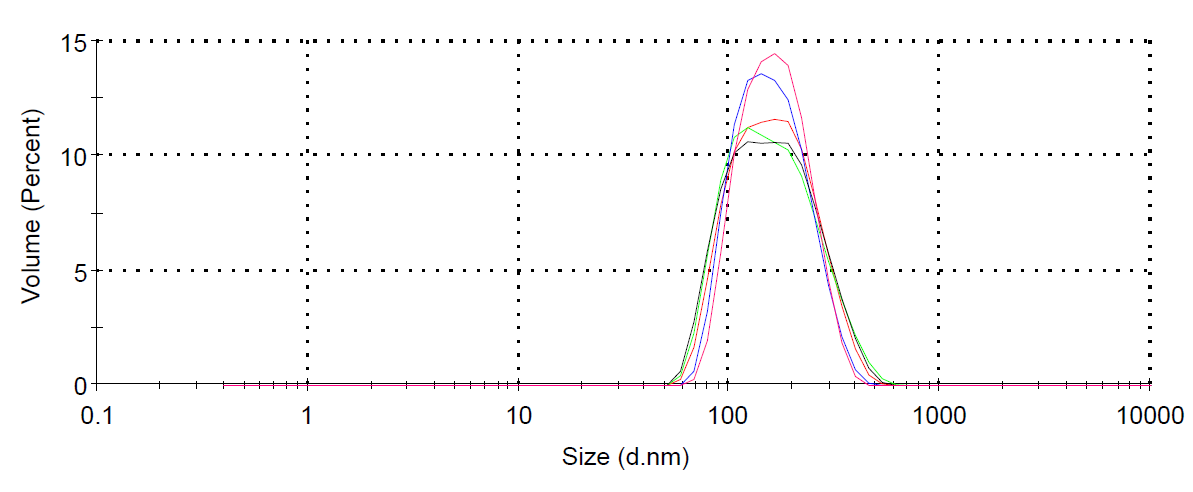


***Figure S3 |*** *Dynamic light scattering of the colloidal fraction of milled fND centrifuged at 3000 rpm for 1 hour. The D50 value of the volume size distribution is 156 nm.*


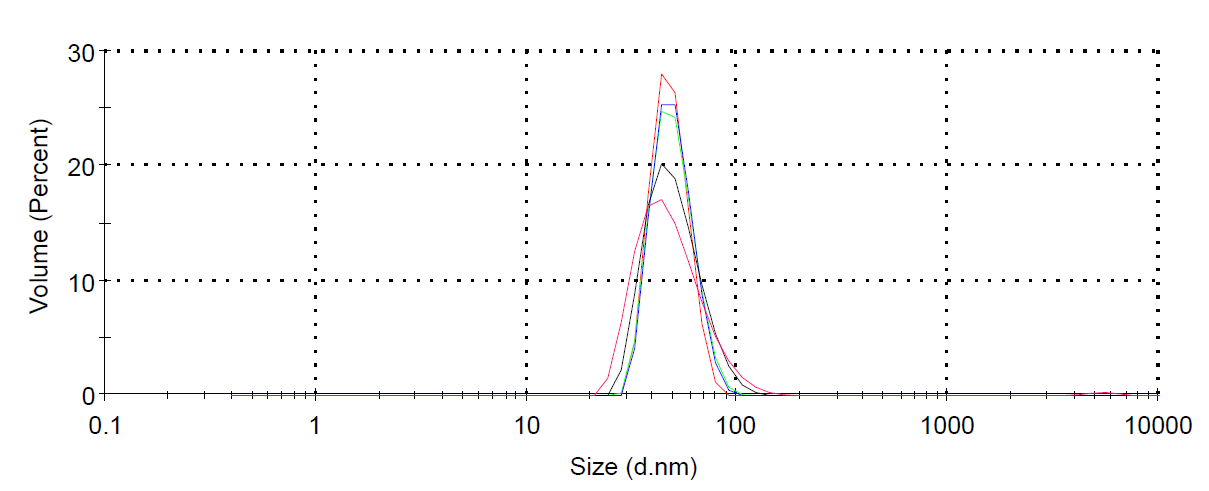


***Figure S4 |*** *Dynamic light scattering of the colloidal fraction of milled fND centrifuged at 15000 rpm for 1 hour. The D50 value of the volume size distribution is 48 nm.*
